# Relationship of SCFAs to Maternal and Child Anthropometric Measurements

**DOI:** 10.3390/ijms26136424

**Published:** 2025-07-03

**Authors:** Małgorzata Szczuko, Natalia Szabunia, Julia Radkiewicz, Dominika Jamioł-Milc, Tomasz Machałowski, Maciej Ziętek

**Affiliations:** 1 Department of Human Nutrition and Metabolomics, Pomeranian Medical University in Szczecin, 70-204 Szczecin, Polanddominika.jamiol.milc@pum.edu.pl (D.J.-M.); 2 Department of Bromatology and Nutritional Diagnostic, Pomeranian Medical University in Szczecin, 70-204 Szczecin, Poland; 3 Department of Perinatology, Obstetrics and Gynecology Pomeranian Medical University in Szczecin, 70-204 Szczecin, Poland; tomasz.machalowski@pum.edu.pl (T.M.); maciej.zietek@pum.edu.pl (M.Z.)

**Keywords:** SCFAs, BCFAs, body mass, BMI, metabolic programming

## Abstract

Short-chain fatty acids (SCFAs) are involved in metabolism and physiological processes. We decided to investigate whether SCFAs are engaged in the metabolic programming of the offspring by the mother’s microbiota, which interact during pregnancy, delivery, and breastfeeding. We decided to determine whether there are correlations between 4-week-old infant feces SCFA concentrations, their weight at birth, and mothers’ anthropometric measurements. The study included 82 women with four-week-old newborns from whom stools were collected. SCFAs were determined using gas chromatography with a flame ionization detector. Correlations were observed between SCFA content in newborns’ feces and mothers’ weight and body mass index (BMI) before delivery and at the time of delivery. In addition, associations were identified between weight gain of pregnant women and SCFAs. Analysis of neonatal data showed associations between fatty acid content and infants’ weight and diet, including breastfeeding. We provide indirect evidence for the association of infant SCFA levels with metabolic programming by maternal gut microbiota metabolites. At the same time, we confirm the influence of increased SCFA levels on higher maternal and neonatal body weight and branched-chain short-chain fatty acids (BCFAs) on neonatal body weight. We provide new preventive and intervention directions for future efforts to improve the health care of pregnant women and their offspring.

## 1. Introduction

In recent years, short-chain fatty acids (SCFAs) have gained considerable attention due to their significant role in metabolism and physiological processes. Anthropometric measurements, which include parameters used to assess nutritional state, can be influenced by SCFAs, which affect carbohydrate metabolism, intestinal barrier tightness, immune system modulation, and appetite control [1,2]. SCFAs have been proven to have many health benefits, such as anti-inflammatory, immunoregulatory, anti-obesity, anti-diabetic, anti-cancer, cardiovascular protective, hepatoprotective, and neuroprotective effects [3]. The substrates for bacterial metabolic processes and the production of SCFAs are food debris undigested by the host’s digestive system. They are mainly polysaccharides. However, the type, amount, and proportion of SCFAs produced by our microbiota mainly depend on the type of our diet and the products we eat.

**Figure 1:**
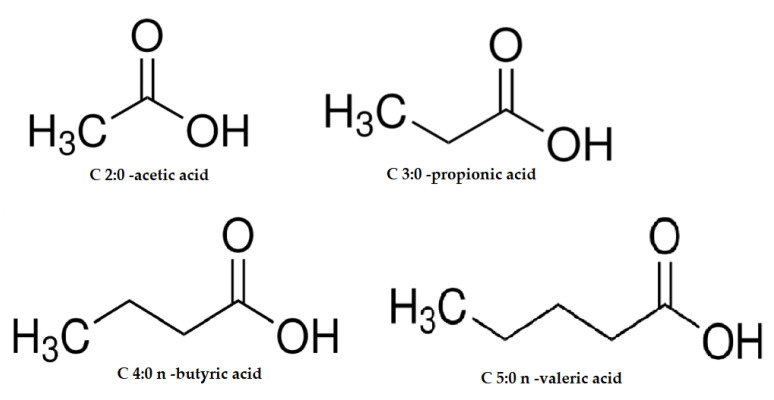
The chemical formulas of the most important SCFAs: acetic acid (C 2:0), propionic acid (C 3:0), butyric acid (C 4:0 n), valeric acid (C 5:0 n).

Acetic acid is one of the main SCFAs produced in the colon. It contributes to increased blood circulation and improved motility in this part of the intestine, and at the cellular level, it also modulates mitochondrial function and fatty acid oxidation [4,5] (Figure 1).

Propionic acid has anti-inflammatory and antimicrobial effects [6]. It can modulate the immune response and can also contribute to maintaining intestinal health by promoting the integrity of the intestinal barrier [7]. Propionic acid salts are effective in inhibiting mold growth, and when combined with lactic and acetic acids, they have been shown to be able to inhibit the growth of *Listeria monocytogenes* [8] (Figure 1).

Butyric acid is an important source of energy for colon cells [9]. It promotes cell division and regenerative and repair processes. It stimulates the local cellular response, maintains the integrity of the intestinal barrier and inhibits the differentiation of tumor cells [10]. It also limits the growth of certain microorganisms and pathogens, such as *Salmonella, Escherichia coli,* and *Campylobacter* [11]. Its protective effects may result from its influence on the proper differentiation and apoptosis of colon cells [12] (Figure 1).

SCFAs can be used by colon cells as a local energy source and participate in many metabolic processes, such as lipid metabolism and glucose homeostasis [13]. They contribute to the regulation of insulin sensitivity and glucose metabolism, potentially influencing the occurrence of some metabolic disorders such as type 2 diabetes or obesity [14]. SCFAs are transported from the intestinal lumen into the host’s blood and are taken up by organs, where they act as substrates or signaling molecules. Therefore, SCFAs produced by the microbiota of the cecum and colon can be detected in hepatic, portal, and peripheral blood [15]. SCFAs are metabolized at three main sites in the body (epithelial cells of the cecum and colon, liver cells, and muscle cells) [14,15].

The process by which the environment affects an organism’s metabolic pathways and functions, especially during crucial developmental stages (such as the prenatal and early postnatal stages), is known as metabolic programming. Studies conducted during pregnancy and early life in animal models suggest that maternally derived SCFAs can cross the placenta, exposing the fetus to them during key developmental periods and affecting critical neurodevelopmental processes. Recent human studies have shown that higher maternal fecal microbiota diversity in the third trimester of pregnancy is associated with a lower incidence of internalizing symptoms in the infant at two years of age. Thus, the mother’s prenatal microbiome is more important for the infant’s neurodevelopment than the infant’s own microbiome [16,17]. SCFAs help regulate increasing insulin resistance during pregnancy and influence the course of gestational diabetes. When lifestyle modification, diet, and physical activity are not enough to achieve the result, supplementation with multi-strain probiotics containing *Bifidobacterium* and *Lactobacillus* can modulate the composition of the gut microbiota and maintain a normal ratio of SCFAs, thereby improving health outcomes [18]. In overweight and obese pregnant women, butyric acid may be particularly important, as it has been observed that its concentration negatively correlates with blood pressure [19]. SCFAs affect the course of pregnancy and the weight of the mother and child over the course of the pregnancy, which is related to the amount and reciprocal ratios of SCFAs.

The concentrations of total SCFAs correlate with anthropometric parameters. The aim of this study is to assess the specificity and relationship of SCFAs with selected maternal and neonatal parameters. Incorporating branched-chain short-chain fatty acid (BCFA) levels with selected anthropometric parameters may reflect early changes in metabolic programming, indicating a potential role of the microbiota.

## 2. Results

The average content of SCFAs is shown in Table 1. It was found that there were some, albeit small, associations between the fecal SCFA content of 4-week-old infants and various anthropometric parameters in both pre-pregnant and post-pregnant women and newborns Table 2.

Positive correlations were found between the weight and BMI of women before and at birth and the content of acetic, propionic, branched butyric, linear valeric, 3,4-methylovaleric, caproic, and heptanoic acids (Table 2). Higher body weight before and at birth was correlated with higher maternal content of these fatty acids. These correlations suggest a potential role for SCFAs in the context of maternal anthropometric parameters. An association between the women’s weight gain during pregnancy and SCFA content was also noted. A positive correlation between these parameters was observed only for acetic acid (0.240), while a negative correlation occurred for all other acids (Table 2). Analyzing the relationship between SCFAs and neonatal weight, it was found that the content of acetic acid and linear butyric acid showed a positive correlation with neonatal weight, which may indicate a relationship between these acids and higher baby weight. There was a negative correlation observed for propionic, branched butyric, branched and linear valeric, 3,4-methylvaleric, caproic, and heptanoic acids (Table 2). Apart from a slight positive correlation of linear butyric acid and branched valeric acid, no statistically significant relationships were observed between the fatty acid content and Apgar scores at 5 min (Table 2). On the other hand, a positive correlation was observed between linear butyric acid content and breastfeeding of newborns from birth. There was also a positive correlation between caproic acid content and feeding newborns.

Using the same program, the paired samples *t*-test was conducted to compare the means of two measurements taken from the same individual and calculate the statistical significance (*p*-value) of the correlations of SCFAs with anthropometric measurements of mothers and children (Table 3). Positive correlations were observed for maternal weight gain during pregnancy with propionic acid and caproic acid and also for children fed with C5n; 3.4 and C7 valeric acid (C 5:0 n), 3.4-methyl valeric acid, and heptanoic acid (Table 3).

**Figure 2:**
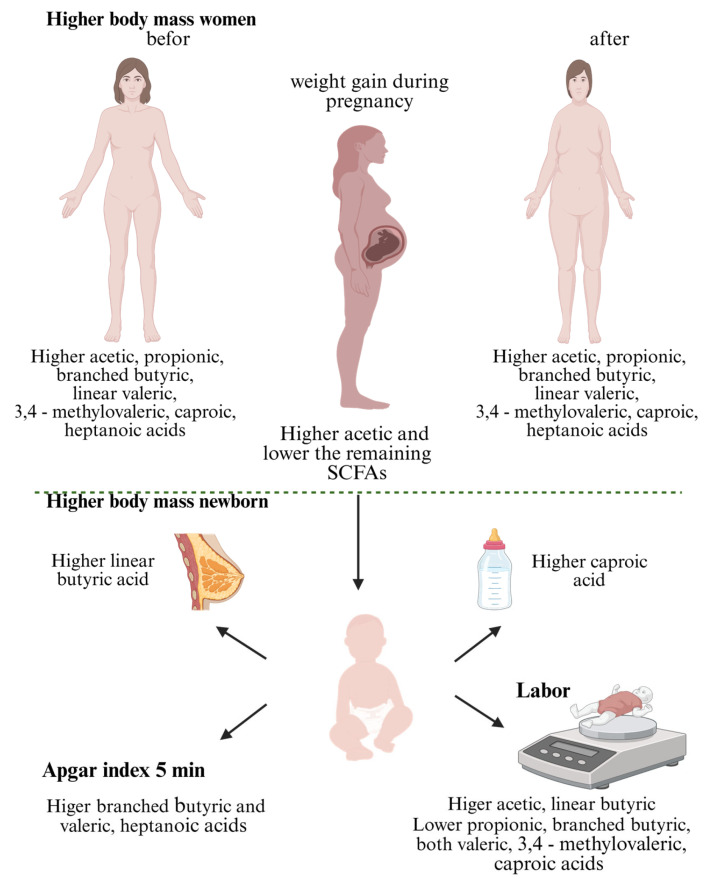
The influence of mother’s SCFAs on newborn weight gain, taking into account the type of feeding (created with BioRender.com, https://app.biorender.com/, accessed on 28 June 2025).

The relationship between maternal SCFA levels and their child’s body mass during pregnancy and delivery, taking into account the method of feeding, is presented in Figure 2.

## 3. Discussion

After analyzing the topic and reviewing the literature, we conclude that this is the first study suggesting a relationship between the occurrence of anthropometric measurements and SCFAs. For this reason, we believe that our work makes a significant contribution to medical knowledge and is innovative and pioneering.

### 3.1. Neonatal SCFAs and Body Weight and BMI of Women

The results of this analysis confirm the role of SCFAs in child weight gain during the perinatal period. We were surprised by the correlations of SCFAs of children with anthropometric measurements of mothers. Body weight and BMI of women before delivery were positively correlated with propionic acid, branched butyric acid, branched valeric acid, linear valeric acid, 3,4-methylvaleric acid, and caproic acid, while a negative correlation was found between acetic acid and linear butyric acid and anthropometric measurements of women before delivery. Higher concentrations of SCFAs, especially butyric acid and propionic acid, may promote increased body weight and body fat; such conclusions were reached by Saipudin et al. when evaluating the Melesian and European populations [20]. However, these correlations may be coincidental due to the long time period, i.e., over about 10 months. Parameters at birth of mothers and newborns seem to more accurately determine the possible relationship of SCFAs with anthropometric parameters. In the case of women, the parameters at birth showed a positive correlation between the weight and BMI of women at birth and propionic acid, branched butyric acid, branched valeric acid, 3,4-methylvaleric, caproic acid, and heptanoic acid, while there was a negative correlation with the other SCFAs studied. These results confirm that an increase in various SCFAs promotes weight gain and higher BMI, which is confirmed by quite a few studies [13,14,20,21]. The correlations between SCFA content and body weight and BMI suggest a potential role for these acids in regulating maternal metabolism and body composition during pregnancy. Acetic acid is used by the liver as an energy source and can affect lipogenesis (fat production), which is important for weight gain. Propionic acid can inhibit lipogenesis in the liver, which can lead to reduced fat gain. Butyric acid improves insulin sensitivity and may affect blood glucose regulation, which is important for preventing excessive weight gain.

### 3.2. Neonatal SCFAs and Neonatal Body Weight and Apgar Score

Analysis of neonatal data revealed associations between SCFA content and anthropometric parameters, as well as the child’s food preferences at birth. There was a positive correlation between neonatal body weight and acetic acid and linear butyric acid and a positive correlation between Apgar score at 5 min and propionic acid, branched butyric acid, linear butyric acid, and branched valeric and linear valeric acids. The results suggest that fatty acid composition can affect child development and improve their condition. In comparison, in a study by Priadarshini and co-authors, researchers found that acetic acid in pregnant women’s serum was associated with women’s weight gain during pregnancy, while maternal acetic and butyric acid levels were not associated with any of the anthropometric measurements in newborns [22].

### 3.3. Neonatal SCFAs and Weight Gain During Pregnancy

In a research paper, Szczuko and co-authors observed a correlation between SCFAs produced by anaerobic carbohydrate fermentation in the large intestine and parameters related to pregnant women’s metabolism and their anthropometric parameters. Since acetic acid makes up the main part of SCFAs (about 37%), correlations were analyzed between the levels of this acid and a group of women who were obese before pregnancy and women of normal weight. Acetic acid may have an impact on anthropometric parameters in both groups of women. Propionic acid was found to correlate positively with weight gain during pregnancy. In the group of women with normal body weight, acetic acid, propionic acid, and linear butyric acid showed a correlation with anthropometric measurements. Negative correlations of linear butyric acid and linear valeric acid with weight gain in women were observed, which suggests that they may reduce weight gain during pregnancy [23]. In an article by Łoniewska et al., the authors showed that the intestinal microbiota producing SCFAs may be able to absorb more energy from the diet, which in some cases can lead to a higher BMI [22]. Malinowska and co-authors conducted a study in which they demonstrated that the synthesis of propionic and butyric acids was positively correlated with several biochemical and anthropometric parameters. All other anthropometric parameters, as well as serum glucose levels and lipid profile, were not significantly correlated with the ability to produce SCFAs [24]. Yamamura and co-authors investigated the relationships between fecal SCFA concentrations and obesity in a relatively large population in Japan [25]. They observed positive linear relationships between BMI and each SCFA subtype. Each fecal SCFA subtype was found to be more prevalent in overweight and obese individuals compared to lean individuals. The study revealed for acetic acid, propionic acid, butyric acid, and total SCFAs that the group with the highest fecal SCFA concentrations had a significantly higher prevalence rate in terms of obesity than the groups with the lowest rate [25]. This suggests that both low SCFA concentrations and high SCFA concentrations may be responsible for deterioration in health. Interestingly, fecal SCFAs were positively associated with the incidence of obesity in Japanese participants, who were less obese, had fewer obesity-associated bacteria (*Roseburia*, *Blautia*, *Akkermansia*, *Eubacterium*, *Coprococcus*, *Fusicatenibacter*), and had a greater number of gut microbiota involved in SCFA production compared to Western countries and other Asian populations. In contrast, the study highlighted that the demonstrated positive association between fecal SCFAs and obesity prevalence is universal among ethnic groups [25]. Among the SCFAs produced by the intestinal microbiota are acetic acid and propionic acid, which are metabolized as substrates for cholesterol synthesis, de novo lipogenesis, adipogenesis, and gluconeogenesis in the liver and white and brown adipocytes, which can lead to fat deposition. All types of fecal SCFAs were significantly higher in the group with higher BMI than in the group with lower BMI, even after correction for the same confounders [26]. Rios-Covian and co-authors (2020) examined fecal concentrations of branched-chain short-chain fatty acids (BCFAs) produced by human intestinal microbiota in relation to age, body mass index (BMI), and diet. It was shown that subjects with morbid obesity (BMI ≥ 40) had significantly higher fecal concentrations of total SCFAs and BCFAs compared to other weight groups [27]. Excluding children, correlation analyses were performed between the molar proportions of fecal isovaleric and isobutyric acids and the sum of both BCFAs with all anthropometric and dietary parameters studied. Isovaleric and isobutyric acids correlated positively between the two. In accordance with this, the group with BMI ≥ 40 showed a significantly higher production of SCFAs, total BCFAs, isobutyric acid, and isovaleric acid, as well as higher proportions of capronate compared to other groups of people with lower BMIs. These results indicate that while it appears that an increase in BMI could have an effect on total SCFA levels, a direct relationship between BCFAs and higher protein intake is not evident in the adult population. Recent studies have shown a link between BCFAs and lipid metabolism, and in this regard, fecal BCFA levels have been found to be higher in hypercholesterolemic individuals compared to normocholesterolemic individuals, with fecal isobutyric acid levels being associated with a poorer serum lipid profile [27]. Szczuko and co-authors, in their study, showed that in a group of women who were overweight or obese before pregnancy, there was a positive correlation between acetic acid and almost all anthropometric and biochemical parameters [23]. Propionic acid was found to correlate positively with weight gain during pregnancy. A similar correlation with SCFAs was found in a group of normal-weight women. It is therefore correlated in everyone, but excess SCFAs, including acetic and propionic acids, can cause overweight and obesity. Negative correlations of C4:0n and C5:0n (linear) with weight gain have been observed [23]. Slizevskaya and co-authors found that obesity could have a significant impact on the metabolites present in the large intestine of children, including SCFAs (formic, acetic, propionic, butyric, and valeric acids) and BCFAs (isobutyric acid and isovaleric acid). The observed differences are mostly negative, as obese children have increased BCFA concentrations compared to normal-weight children [28].

Ostrowska and co-authors examined the effect of a high-fat diet on fecal SCFA concentrations. Propionic and butyric acids had a significant negative association with waist circumference in men. Valeric acid was also shown to have the same significant negative association with fat and pendulous adipose tissue in women [29]. Elevated fecal SCFA concentrations are a risk factor for cardiometabolic diseases, overweight, and obesity. These findings may be explained by the low absorption of SCFAs in intestinal epithelial cells, which may have been linked to a high-fat diet and the presence of chronic inflammation associated with overweight and obesity [29]. The above-mentioned research studies showed various correlations and relationships between SCFAs and anthropometric measurements, and the results were not always conclusive. In more cases, other authors have shown a positive correlation of SCFAs with anthropometric measurements and increased fecal SCFA concentrations in individuals with higher BMI, higher body weight, overweight, or obesity. Their results are in accordance with the findings of our own study. Women’s weight gain during pregnancy was negatively correlated with propionic and caproic acid, which means that their higher fecal concentrations may be associated with lower weight gain in pregnant women. In the case of newborns, analysis of the data revealed associations between SCFA content and anthropometric parameters, as well as the child’s food preferences at birth. There was a statistically significant positive correlation between neonatal feeding and concentrations of linear valeric acid, 3,4-methylvaleric acid, and heptanoic acid, suggesting a higher content of these SCFAs in children who were additionally fed. Thus, supplemental feeding may promote faster child weight gain. Reduced systemic inflammation, increased energy expenditure, improved insulin sensitivity, satiety stimulation, mitochondrial function, blood pressure reduction, appetite suppression, and improved cognitive function from a variety of neurologic diseases are all caused by SCFAs [30,31].

It is important to acknowledge the observational nature of our research, which has resulted in significant challenges when attempting to interpret the findings. This is due to the fact that there are a multitude of factors capable of altering the quantity and quality of SCFAs in the stool, including diet (intake of fiber, type of carbohydrates, fat, and protein consumed). The composition of the intestinal microbiota is crucial for SCFA production, as a change in the composition of the microbiome leads to reduced production of these acids. Furthermore, it is important to note that certain gastrointestinal diseases, including irritable bowel syndrome, Crohn’s disease, ulcerative colitis, and other inflammatory bowel diseases, have the capacity to influence SCFA production. Moreover, the utilization of antibiotics has been demonstrated to disrupt the equilibrium of the gut microbiota, thereby impeding the capacity of bacteria to produce SCFAs. The hypothesis that an individual’s genotype can influence the composition of the microbiome and, consequently, the capacity to produce SCFAs remains to be substantiated through further research.

### 3.4. Metabolic Role of SCFAs

SCFAs, such as acetic acid, propionic acid, and butyric acid, play an important metabolic role, affecting various biochemical processes in human bodies, including insulin sensitivity, lipid metabolism, and energy homeostasis [19,23].

SCFAs have been demonstrated to have a beneficial effect on insulin sensitivity, which is pivotal in the regulation of blood glucose levels and the prevention of type 2 diabetes. SCFAs, notably butyric acid, engage with G-protein receptors such as GPR41 and GPR43, which are expressed in pancreatic beta cells, adipocytes, intestines, and tissues of other organs. The activation of these receptors has been shown to influence signaling pathways, thereby enhancing cellular sensitivity to insulin. Butyric acid, through its action on the intestinal microbiota, can increase the production of incretin hormones (e.g., GLP-1), which stimulate insulin secretion in response to a meal. Consequently, this contributes to the regulation of blood glucose levels. SCFAs have been shown to possess anti-inflammatory properties, which can influence insulin sensitivity. In conditions associated with inflammation (a common occurrence in cases of obesity and insulin resistance), the reduction in inflammatory cytokines (e.g., TNF-α, IL-6) that hinder insulin action can be attributed to SCFA production [19].

In summary, SCFAs have important effects on a number of metabolic processes in the body, including insulin sensitivity, lipid metabolism, and energy homeostasis. By acting on the gut microbiota, SCFAs improve insulin sensitivity, promote lipid metabolism, and regulate the body’s energy balance. Their beneficial effects on metabolic health, particularly during pregnancy, make them key mediators in the prevention and treatment of metabolic disorders such as type 2 diabetes, obesity, and cardiovascular disease. Regular consumption of a diet rich in fiber during pregnancy, which is fermented into SCFAs by the gut microbiota, may have significant health benefits for the newborn.

### 3.5. Limitations

Although we obtained promising results that may have significant clinical implications, the study has its limitations: a small group of participants, various factors that may influence the disorders (diet changes, physical activity, antibiotic therapy), and the lack of an ability to validate the obtained dietary data. The sample size should be increased to mitigate the impact of potential random dependencies.

## 4. Materials and Methods

### 4.1. Study Group

Eighty-two women with newborns participated in the study, with each pregnancy being a single pregnancy. All the patients gave birth via cesarean section, and all the women had similar dietary habits. All the patients belonged to the Caucasian ethnic group; they were homogeneous in terms of ethnicity. When selecting patients, socioeconomic factors were taken into account; the patients did not have any addictions (smoking, alcohol), had secondary or higher education, and had an intermediate material status. The anthropometric measurements were obtained using the MS 4971 Charden (Jawag, Guozhong, Taichung, Taiwan) medical column scale. The instrument possesses a weighing range of 100 g, facilitating precise body mass index determination (BMI) and height assessment ranging from 60 to 210 cm. The Apgar scale was utilized to evaluate the health status of the newborn infant in the initial minutes of life. It evaluates five parameters that are important for the functioning of the baby’s body: heart rate, respiratory effort, muscle tone, reflex response, and skin color. Each of these five parameters is scored on a scale of 0 to 2 points. The total sum of the points from all the parameters gives the final score, which can range from 0 to 10 points. The interpretation of the results is as follows: Scores ranging from 8 to 10 points indicate that the newborn is in good condition and does not require special medical intervention. Scores ranging from 5 to 7 points indicate a necessity for assistance, which may take the form of respiratory stimulation or oxygen therapy. Finally, a score of 0–4 points indicates an immediate requirement for medical intervention, including oxygen administration, cardiac massage, or other remedial measures. The average age of the women studied was 31.17 ± 4.51 years. Pregnancy weight gain averaged 14.24 ± 7.02 kg. The mean body mass index (BMI) before and at delivery was 24.56 ± 6.05 and 29.57 ± 5.615, respectively. Table 4 shows the anthropometric measurements of the women and newborns studied. Newborns were examined at their first month of life. The study group of newborns included 37 boys and 45 girls. All the newborns were breastfed from birth, and 44 of the 82 were supplementally fed. The average weight of the newborns at birth was 3286.339 ± 394.79 g (Table 4).

### 4.2. Determination of SCFAs in a Stool Sample

All study participants’ parents were asked to collect a stool sample into a screw-capped collection container and were required not to use laxatives or change the diet. Following stool collection, the samples were promptly frozen at −20 °C and sent to our lab on ice. They were then kept at −80 °C until the analyses were completed. Six months following the collection of the fecal sample, we conducted the analysis. Newborns’ feces samples were collected after 4 weeks of age at the time of the pediatrician’s follow-up visit. The feces samples were stored at −80 °C, and then SCFAs were determined using gas chromatography (GC). A fecal sample of 0.5 g suspended and mixed in 5 mL of distilled water was used to determine SCFAs. The SCFAs examined were acetic acid (C 2:0), propionic acid (C 3:0), isobutyric acid (C 4:0 i), butyric acid (C 4:0 n), isovaleric acid (C 5:0 i), valeric acid (C 5:0 n), caproic acid (C 6:0 n), and heptanoic acid (C 7:0). The supernatant was filtered (Ø 400 µm filter) [23]. Chromatographic analyses were performed using a GC 1260 A system from Agilent Technologies (Cheadle, UK) with a flame ionization detector (FID). A silica gel capillary column with a free fatty acid phase (DB-FFAP, 30 m × 0.53 mm × 0.5 um) was used. The injection volume was 1 μL, and the time to perform a single analysis was 17.5 min. Fatty acids were identified by comparing their retention times with those of commercially available standards [23].

### 4.3. Statistical Analysis

Statistical analyses were performed using Microsoft Excel (Analysis ToolPak add-on) and Statistica 13.3 (Statsoft, Krakow, Poland). Descriptive statistics of anthropometric measurements of the women studied (Table 4) and fecal SCFA contents in newborns (Table 1) were analyzed using Microsoft Excel. SCFA content was then correlated with anthropometric measurements in women and newborns, and a correlation matrix was generated using Microsoft Excel (Figure 2). Using the same program, paired *t*-tests were performed with two samples for the mean to calculate *p*-values. The level of statistical significance was a *p*-value set at 0.05.

## 5. Conclusions

The analysis of the results of the study showed correlations between the content of SCFAs in the feces of infants and the anthropometric parameters of pregnant women and their offspring. It was observed that the SCFAs of the newborns were related to the mothers’ weight before delivery, their BMI, and their growth during pregnancy, as well as the weight of the newborns. The short-chain fatty acid profile in the newborn may reflect the mother’s metabolic status during pregnancy. This suggests a potential role of the maternal microbiota and its metabolites in metabolic programming already during the perinatal period. In conclusion, this work sets preventive and interventional directions for future efforts to improve health care in this area and highlights the important role of SCFAs in maternal and child health during the perinatal period, with connotations for later stages of the offspring’s life. The variability in SCFAs, changes in dietary habits, and physical activity have an impact on the health of the mother and child, proper colonization by microbiota, influence on morbidity, and the proper development of the immune system. Strict control of body mass and a diet that includes better proposals for individual fatty acids, known as a residual diet, allow for better development of the metabolic system and the aforementioned immune system.

## Figures and Tables

**Table 1:** Fecal SCFA contents in newborns.

Fatty Acids	AVG (mol/g Feces Dry Weight)	SD	Median	Standard Error
C2:0	55.02	7.329	55.755	1.105
C3:0	10.375	2.125	10.692	0.32
C4i	4.974	1.212	5.065	0.183
C4n	4.473	2.13	3.947	0.321
C5i	3.128	0.931	2.926	0.14
C5n	1.156	1.781	0.467	0.268
3,4-methylovaleric	1.318	1.51	0.747	0.228
C6:0	18.52	4.416	19.196	0.666
C7:0	1.037	1.396	0.471	0.21

The chemical names of SCFAs are as follows: acetic acid (C 2:0), propionic acid (C 3:0), isobutyric acid; (C 4:0 i), butyric acid (C 4:0 n), isovaleric acid (C 5:0 i), valeric acid (C 5:0 n), caproic acid (C 6:0 n), and heptanoic acid (C 7:0); AVG: average value of an SCFA stool data (mol/g feces dry weight); SD: standard deviation.

**Table 2:** SCFA correlation matrix with anthropometric measurements of mothers and children.

	Pre-Delivery BW of Women	BW of Women at Delivery	Pre-Delivery BMI of Women	BMl of Women at Delivery	Gestational Weight Gain	Birth Weight	Apgar Score at 5 min	C2:0	C3:0	C4i	C4n	C5i	C5n	3,4-Methylo valeric	C6:0	C7:0
Pre-delivery BW of women	1	0.940	0.962	0.962	−0.156	−0.232	−0.087	−0.202	0.127	0.076	−0.034	0.034	0.105	0.100	0.141	0.143
BW of women at delivery	0.940	1	0.921	0.959	0.165	−0.171	−0.127	−0.107	0.119	0.028	−0.051	−0.063	0.048	0.033	0.094	0.080
Pre-delivery BMI of women	0.962	0.921	1	0.942	−0.049	−0.174	−0.114	−0.166	0.100	0.079	−0.048	0.041	0.072	0.068	0.131	0.131
BMI of women at delivery	0.962	0.959	0.942	1	0.212	−0.212	−0.131	−0.066	0.107	0.036	−0.073	−0.041	0.018	0.003	0.068	0.052
Gestational weight gain	−0.156	0.165	−0.049	0.212	1	0.203	−0.050	0.240	−0.072	−0.036	−0.063	−0.259	−0.173	−0.200	−0.078	−0.169
Birth weight	−0.232	−0.171	−0.174	−0.212	0.203	1	0.021	0.193	−0.071	−0.145	0.151	−0.240	−0.067	−0.142	−0.153	−0.126
Apgar score at 5 min	−0.087	−0.127	−0.114	−0.131	−0.050	0.021	1	−0.034	0.083	0.085	0.135	0.113	0.012	−0.029	−0.073	−0.058
C2:0	−0.202	−0.107	−0.166	−0.066	0.240	0.193	−0.034	1	−0.071	−0.702	−0.083	−0.605	−0.718	−0.751	−0486	+0.737
C3:0	0.127	0.119	0.100	0.107	−0.072	−0.071	0.083	−0.071	1	0.073	−0.576	−0.023	−0.299	−0.266	0.215	−0.328
C4i	0.076	0.028	0.079	0.036	−0.036	−0.145	0.085	−0.702	0.073	1	−0.214	0.130	0.225	0.269	0.672	0.245
C4n	−0.034	−0.051	−0.048	−0.073	−0.063	0.151	0.135	−0.083	−0.576	−0.214	1	0.254	0.516	0.480	−0.590	0.488
C5i	0.034	−0.063	0.041	−0.041	−0.259	−0.240	0.113	−0.605	−0.023	0.130	0.254	1	0.757	0.797	−0.166	0.741
C5n	0.105	0.048	0.072	0.018	−0.173	−0.067	0.012	−0.718	−0.299	0.225	0.516	0.757	1	0.974	−0.182	0.982
3,4-methylovaleric	0.100	0.033	0.068	0.003	−0.200	−0.142	−0.029	−0.751	−0.266	0.269	0.480	0.797	0.974	1	−0.143	0.979
C6:0	0.141	0.094	0.131	0.068	−0.078	−0.153	−0.073	−0.486	0.215	0.672	−0.590	−0.166	−0.182	−0.143	1	−0.124
C7:0	0.143	0.080	0.131	0.052	−0.169	−0.126	−0.058	−0.737	−0.328	0.245	0.488	0.741	0.982	0.979	−0.124	1

The chemical names of SCFAs are as follows: acetic acid (C 2:0), propionic acid (C 3:0), isobutyric acid (C 4:0 i), butyric acid (C 4:0 n), isovaleric acid (C 5:0 i), valeric acid (C 5:0 n), caproic acid (C 6:0 n), and heptanoic acid (C 7:0). SCSA correlations index: 0.2 weak correlation; 0.3–0.5 sufficient correlation; 0.6–0.7 moderate correlation; 0.8–0.9 very strong correlation.

**Table 3:** Statistical significance of the relationship of SCFAs with anthropometric measurements of mothers and children.

	C2:0	C3:0	C4i	C4n	C5i	C5n	3,4-Methylvaleric	C6:0	C7:0
Pre-delivery BW of women	*p* = 5.95	*p* = 2.39	*p* = 9.91	*p* = 1.18	*p* = 3.60	*p* = 9.39	*p* = 1.06	*p* = 6.71	*p* = 8.03
BW of women at delivery	*p* = 1.71	*p* = 6.69	*p* = 5.05	*p* = 6.25	*p* = 2.29	*p* = 7.11	*p* = 7.90	*p* = 1.27	*p* = 5.89
Pre-delivery BMI of women	*p* = 7.29	*p* = 8.39	*p* = 6.62	*p* = 6.56	*p* = 6.11	*p* = 5.56	*p* = 5.60	*p* = 1.19	*p* = 5.51
BMI of women at delivery	*p* = 4.02	*p* = 3.31	*p* = 1.62	*p* = 8.82	*p* = 1.13	*p* = 1.20	*p* = 1.18	*p* = 6.98	*p* = 4.51
Gestational weight gain	*p* = 3.77	*p* = 0.006 *p* < 0.05	*p* = 1.95	*p* = 1.19	*p* = 2.18	*p* = 3.18	*p* = 3.76	*p* = 0.001 *p* < 0.05	*p* = 1.37
Birth weight	*p* = 7.71	*p* = 5.06	*p* = 4.72	*p* = 4.42	*p* = 4.63	*p* = 4.46	*p* = 4.52	*p* = 6.03	*p* = 4.48
Apgar score at 5 min	*p* = 5.51	*p* = 0.06	*p* = 1.22	*p* = 4.27	*p* = 2.31	*p* = 1.09	*p* = 1.09	*p* = 2.11	*p* = 2.69
Breastfeeding	*p* = 2.48	*p* = 3.04	*p* = 4.45	*p* = 6.27	*p* = 8.55	*p* = 0.5	*p* = 0.15	*p* = 3.79	*p* = 0.78
Supplementary feeding	*p* = 2.34	*p* = 1.66	*p* = 3.67	*p* = 5.02	*p* = 8.03	*p* = 0.02 *p* < 0.05	*p* = 0.002 *p* < 0.05	*p* = 2.16	*p* = 0.03 *p* < 0.05

The chemical names of SCFAs are as follows: acetic acid (C 2:0), propionic acid (C 3:0), isobutyric acid (C 4:0 i), butyric acid (C 4:0 n), isovaleric acid (C 5:0 i), valeric acid (C 5:0 n), caproic acid (C 6:0 n), and heptanoic acid (C 7:0). Statistical significance threshold is *p* < 0.05.

**Table 4:** Anthropometric measurements of the women studied.

Parameters	AVG	SD	Median	Min–Max
Age [years]	31.17	4.49	30.5	22–40
Weight before delivery [kg]	68.28	17.59	65	40–126
Weight at birth [kg]	82.19	16.55	80	52–142
BMI before delivery [kg/m^2^]	24.56	6.05	22.73	15.94–48.01
BMI at delivery [kg/m^2^]	29.57	5.62	28.4	19.84–54.11
Weight gain [kg]	14.24	7.01	13	−2–37
Weight at birth [g]	3286.34	394.79	3285	2090–4390
Apgar score at 5 min.	9.78	0.0576	10	7–10

AVG: average value of the verse; SD: standard deviation.

## Data Availability

Dataset is available on request from the authors.

## Abbreviations

BCFA	branched-chain short-chain fatty acid
BMI	body mass index
C 2:0	acetic acid
C 3:0	propionic acid
C 4:0 i	isobutyric acid
C 4:0 n	butyric acid
C 5:0 i	isovaleric acid
C 5:0 n	valeric acid
C 6:0 n	caproic acid
C 7:0	heptanoic acid
SCFA	short-chain fatty acid
